# Leveraging genomic information to predict environmental preferences of bacteria

**DOI:** 10.1093/ismejo/wrae195

**Published:** 2024-10-03

**Authors:** Josep Ramoneda, Michael Hoffert, Elias Stallard-Olivera, Emilio O Casamayor, Noah Fierer

**Affiliations:** Department of Ecology and Complexity, Center of Advanced Studies of Blanes (CEAB), Spanish Research Council (CSIC), Blanes, Spain; Cooperative Institute for Research in Environmental Sciences (CIRES), University of Colorado, Boulder, Colorado, United States; Department of Ecology and Evolutionary Biology, University of Colorado, Boulder, CO, United States; Department of Ecology and Evolutionary Biology, University of Colorado, Boulder, CO, United States; Department of Ecology and Complexity, Center of Advanced Studies of Blanes (CEAB), Spanish Research Council (CSIC), Blanes, Spain; Cooperative Institute for Research in Environmental Sciences (CIRES), University of Colorado, Boulder, Colorado, United States; Department of Ecology and Evolutionary Biology, University of Colorado, Boulder, CO, United States

**Keywords:** environmental preferences, microbial adaptation, genome-based models, machine learning, bacterial cultivation, environmental factors, uncultured bacteria, microbial ecology

## Abstract

Genomic information is now available for a broad diversity of bacteria, including uncultivated taxa. However, we have corresponding knowledge on environmental preferences (i.e. bacterial growth responses across gradients in oxygen, pH, temperature, salinity, and other environmental conditions) for a relatively narrow swath of bacterial diversity. These limits to our understanding of bacterial ecologies constrain our ability to predict how assemblages will shift in response to global change factors, design effective probiotics, or guide cultivation efforts. We need innovative approaches that take advantage of expanding genome databases to accurately infer the environmental preferences of bacteria and validate the accuracy of these inferences. By doing so, we can broaden our quantitative understanding of the environmental preferences of the majority of bacterial taxa that remain uncharacterized. With this perspective, we highlight why it is important to infer environmental preferences from genomic information and discuss the range of potential strategies for doing so. In particular, we highlight concrete examples of how both cultivation-independent and cultivation-dependent approaches can be integrated with genomic data to develop predictive models. We also emphasize the limitations and pitfalls of these approaches and the specific knowledge gaps that need to be addressed to successfully expand our understanding of the environmental preferences of bacteria.

## What are environmental preferences?

All microorganisms have an environmental preference, and these preferences vary depending on the taxa in question. The term environmental preference encapsulates both the range of conditions that support the growth of a certain taxon as well as the specific conditions where growth is maximized (i.e. a growth optimum and range). In essence, environmental preferences are the growth responses of taxa across environmental gradients. Quantifying environmental preferences is therefore often the key to determining when and where any given microbial taxon will grow [1], a long-standing challenge in microbial ecology. Despite the importance of understanding the environmental preferences of microorganisms, we only have empirical information on environmental preferences for a small subset of microbial diversity [2], emphasizing the importance of innovative approaches that address this knowledge gap, particularly for the many taxa that remain uncultured [3]. Even for well-studied isolates, some key environmental preferences such as growth responses across gradients in moisture availability are rarely quantified. Also, environmental preferences generally reflect a complex combination of phenotypic traits, such as the capacity to maintain pH homeostasis, or to maintain cellular integrity under elevated temperatures, and many of these traits are not well understood. With this Perspective piece, we present the current gaps and biases in our understanding of bacterial environmental preferences, discuss the empirical approaches that can be used to infer environmental preferences across a broad range of taxa, and showcase the utility of genome-based approaches for expanding our understanding of bacterial environmental preferences.

## Why is it important to quantify the environmental preferences of bacteria?

Bacteria cope with a broad range of growth-limiting environmental factors that contribute to their ecological niches [4]. For example, salinity is often a primary determinant of bacterial distributions in aquatic environments [5, 6], pH exerts a strong control over soil bacterial community structure [7], and changes in oxygen levels can contribute to compositional shifts in the human gut microbiome [8]. Improving our quantitative understanding of bacterial environmental preferences, both within and between species, would provide a range of benefits to the field of microbial ecology. For example, bacterial cultivation success could be improved if we knew more precisely the set of conditions that are most likely to maximize a target taxon’s growth [9], and the efficacy of human probiotics could be improved by tailoring the consortia for efficient growth under the specific pH or oxygen conditions of the gut [10]. In agriculture, bacterial inoculants often fail to deliver promised benefits to plant–soil systems, where *a priori* knowledge of the conditions that best support growth could improve the effectiveness and persistence of inoculants [11]. Fundamentally, descriptions of the genes or genomic attributes underlying environmental adaptations, and how these adaptations relate to biogeographical patterns, would improve predictive modeling of bacterial distributions across time and space [12]. This is particularly relevant in the context of global change, where it is often difficult to predict *a priori* how shifts in environmental conditions will alter bacterial distributions in ecosystems. For example, understanding how sea level rise and the associated salinization of coastal wetlands may impact bacterial communities requires prior knowledge of taxon-specific salinity preferences [13]. Similarly, predicting the impacts of climate change on soil bacterial communities would benefit from knowledge of both taxon-specific moisture and temperature preferences. Persistent and important challenges in microbial ecology could thus be addressed by improving our current understanding of the environmental preferences of bacteria and the adaptations that underly those preferences.

## Importance of environmental preferences in different microbial systems

**Figure 1:**
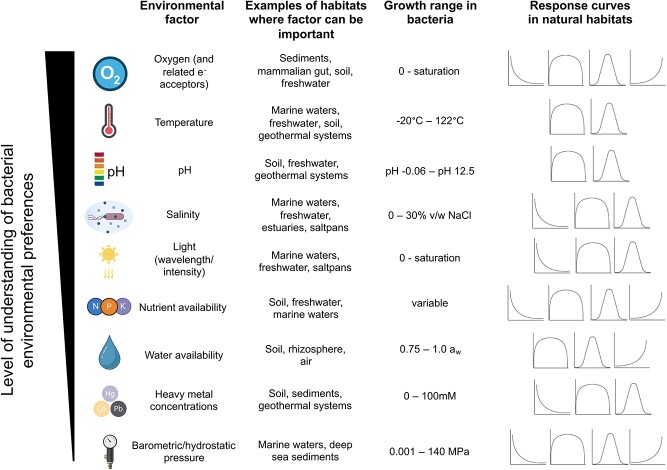
Growth range and bacterial growth responses to the environmental factors that are relevant to bacterial environmental adaptation across habitats. Environmental factors are sorted by decreasing degree of knowledge of bacterial environmental preferences.

Environmental factors influence bacterial adaptation and growth by limiting access to electron donors and acceptors (oxygen availability, pH), by affecting the efficiency of energy-yielding reactions (temperature, water availability, light availability), or by imposing physical constraints or toxicity to metabolic functions (pH, salt and heavy metal concentrations, barometric, and hydrostatic pressure) (Fig. 1). Although biotic interactions can also limit bacterial growth and there are ongoing efforts to predict biotic interactions from genomic information [14, 15], we restrict our discussion here to physicochemical factors. The importance of specific environmental factors on bacterial growth will obviously vary depending on the system in question. For example, as the pH of marine waters is relatively invariant and generally around pH 8, pH is a less important factor for structuring marine bacterial communities compared to temperature and light availability, which display strong latitudinal and vertical gradients and are known to select for diverse bacterial ecotypes (e.g. planktonic cyanobacterial *Prochlorococcus*; [14]). In contrast, pH is an important driver of bacterial community structure across both soils and freshwater ecosystems ([7, 15]; Fig. 1). Likewise, water availability is limiting for bacterial growth in highly saline systems [16], skin [17], and soil environments [18], but less important in most marine, freshwater, and gut systems (Fig. 1).

Environmental preferences define the range of environmental conditions where a particular microbe can grow: growth is commonly constrained by one or multiple environmental extremes, and what constitutes “extreme” varies amongst organisms. For example, high concentrations of heavy metals, as can be found in some geothermal systems [19], can inhibit growth due to toxicity [20]. Most bacterial taxa exhibit decreasing growth in response to elevated heavy metal concentrations, where heavy metal-adapted taxa display tolerance but not increased growth ([21]). In contrast, bacteria generally exhibit a constrained optimum for factors such as temperature or pH (Fig. 1), which can often have interactive effects, so that for example the pH optimum of an organism often depends on temperature [22]. A more nuanced description of the growth responses of bacteria across environmental gradients would thus improve our understanding of the habitability limits of bacteria.

## Biases in our understanding of bacterial environmental preferences

**Figure 2:**
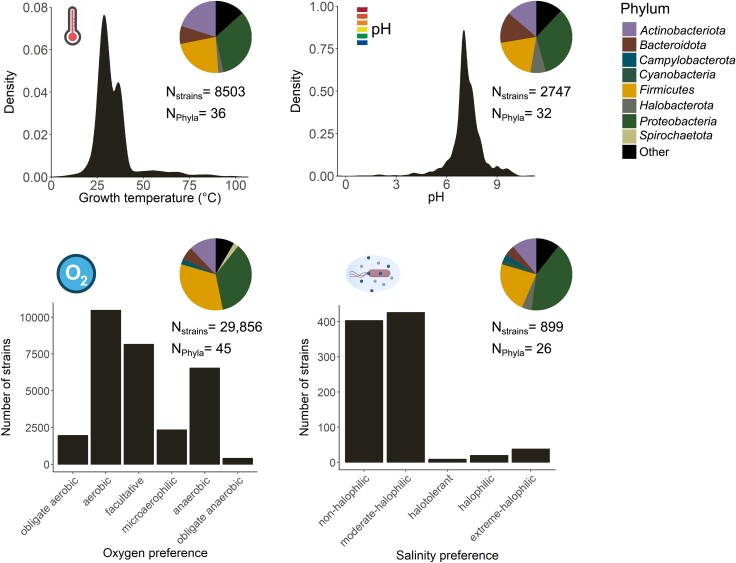
Current quantitative and taxonomic distribution of environmental preferences among cultivated bacteria. The analysis is based on the bacterial phenotypic trait compilation from reference [29].

Characterization of bacterial environmental preferences traditionally relies on laboratory-based quantification of growth responses of isolates along well-defined gradients in growth-limiting factors such as oxygen concentrations [23], temperature [24], moisture [25], pH [26], salinity [27], or barometric pressure [28]. For example, at previous study tested the growth responses of 23 bacterial strains along a moisture gradient in artificial soil microcosms to determine their moisture preferences [25]. This approach made it possible to identify the functional traits associated with moisture preference in these taxa, in addition to precisely describing taxon-specific moisture tolerances and optima. Bacterial cultivation allows us for the precise tuning of the environmental gradient, the control of confounding factors, and an accurate quantification of bacterial growth responses. Although bacterial cultivation has expanded our understanding of bacterial environmental preferences, the availability of information on bacterial environmental preferences is often limited (Fig. 2), with growth responses across environmental gradients remaining poorly characterized for certain environmental factors and taxonomic groups. For example, one of the most complete syntheses of bacterial phenotypic trait information contains information on the oxygen availability preferences of ~30 000 bacterial strains including members 45 different phyla [29], whereas information on salinity preferences is only available for ca. 900 strains covering 26 different phyla (Fig. 2). This information is mostly available for a narrow range of environmental preferences and for taxa that grow well under “standard” culturing conditions (Fig. 2).

The reliance on cultivation has led to multiple biases in our understanding of environmental preferences. First, uncultivated taxa dominate in many environments [30], but their environmental preferences remain largely uncharacterized. This means the taxonomic and phylogenetic breadth of environmental preference information is heavily biased towards the relatively narrow subset of bacterial taxa that are readily culturable. Second, bacterial cultivation has historically followed standardized growth conditions where isolates are grown at temperatures around 30°C, neutral pH, and aerobic conditions ([31]; Fig. 2). Although there are many notable exceptions, the methodological biases associated with “standard” culturing conditions limit the empirical range for which environmental preference information is available. Third, particular environmental factors require specialized equipment for their manipulation, as in the case of hydrostatic pressure, so information on bacterial responses to these factors is very limited. Finally, testing bacterial growth responses along environmental gradients under-controlled laboratory conditions is a laborious task, which becomes particularly daunting for taxa that are difficult to maintain in culture. These biases largely restrict our current understanding of bacterial environmental preferences to the readily culturable and often human-associated members of the *Actinobacteriota*, *Bacteroidota*, *Firmicutes*, and *Proteobacteria* (Fig. 2). To broaden our understanding of bacterial environmental preferences, we need to complement cultivation-dependent approaches for quantifying environmental preferences with cultivation-independent approaches.

## Quantification of bacterial environmental preferences using culture-independent approaches

Although novel cultivation approaches make it possible to isolate a broader diversity of taxa [32], it remains challenging to expand the taxonomic breadth of taxa with environmental preference information using culture-based methods alone. Despite the inherent limitations, culture-independent methods have the potential to expand our understanding of the environmental preferences of currently uncharacterized taxa [33]. The analysis of biogeographic patterns is an approach that can provide novel information on bacterial environmental preferences [34, 35]. One key assumption of such an approach is that abundance distributions reflect the growth responses of bacterial taxa and that the environmental preferences of taxa should be reflected by taxon-specific changes in abundance across environmental gradients. While nontrivial, the validity of this assumption could be explicitly tested by using cultivation-independent methods to quantify *in situ* growth rates of specific taxa across environmental gradients [36, 37]. Of course, there are reasons this assumption may not hold as local conditions can often lead to biomass accumulation in areas that do not support optimal growth (e.g. in hypersaline systems, [38]), and certain taxa can produce resistant structures that allow them to persist under adverse conditions [39]. It can also be challenging to ascribe changes in bacterial abundances to a single environmental factor, as multiple environmental factors often co-vary in the field, and biotic controls over bacterial biomass, such as viral predation, are not homogenously distributed in space [40]. Yet, environmental preferences can often be inferred from observed biogeographic distributions in terrestrial and marine ecosystems without the need for laboratory-based testing of growth responses [36]. For example, the distribution of bacterioplankton across salinity gradients in the Baltic Sea can be used to infer the salinity preferences of a broad range of aquatic taxa [6], many of which remain uncultured.

**Figure 3:**
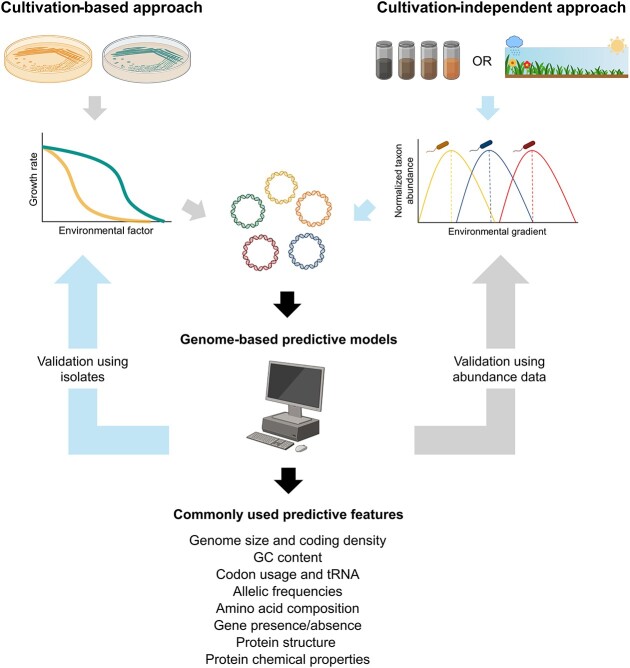
Empirical and predictive approaches to expand our understanding of microbial environmental preferences. Expanding our current understanding of bacterial environmental preferences requires the integration of culture-based and culture-independent approaches that leverage the genomic information that is increasingly available for bacteria. We provide a list of genomic and proteomic features that are commonly used for the prediction of environmental preferences in bacteria.

Culture-independent methods can also be applied in experimental systems, where environmental conditions are manipulated using controlled experiments and changes in bacterial community structure to a given environmental factor are tested (e.g. in soil or aquatic microcosms, [41]). For example, shade-out experiments have been used to identify biofilm-dwelling bacteria with low, intermediate, and high light availability preferences in laboratory-based freshwater microcosms [42]. The upside of such culture-independent approaches is that they can cover a broader taxonomic diversity and allow for the measurement of taxon-specific preferences under environmental conditions that are more representative than culture-based assays. However, manipulation of specific environmental conditions can often lead to confounding effects. For example, increasing moisture generally reduces oxygen availability in soil, so experimental manipulations of soil water content are likely to concurrently affect soil oxygen levels that can impact bacterial growth responses [43]. There is clearly no ideal method for inferring environmental preferences. Rather, culture-based and culture-independent approaches are complementary, and the integration of these approaches can be more valuable than relying on a single approach (Fig. 3).

## Can we use genomic information to infer bacterial environmental preferences?

Novel genomic information is being generated at a much faster pace than information on environmental preferences. While bacterial responses to the environment can be highly plastic, ranges in environmental preferences can be potentially inferred from genomic data. Such analyses could be based on general genomic features without annotation (e.g. genome size, GC content, and k-mer-based nucleotide frequencies; [44, 45]), amino acid composition ([46]), or from specific gene and protein information such as the GC content of particular genes ([47]), gene presence/absence ([48]), the relative abundances of particular genes per genome [49], gene variants [50], or inferred protein properties ([5]) (Fig. 3). Note that not all environmental preferences necessarily require whole-genome information to be predicted, as preferences can often exhibit a phylogenetic signal [51], allowing for predictions based on phylogenetic or taxonomic information alone. For example, thermophilic and hyper-thermophilic bacteria are often taxonomically distinct, even at the phylum level of resolution [52]. Likewise, since related taxa often share similar oxygen requirements [53], oxygen preferences of novel taxa can, in some cases, be inferred from related taxa that have been well characterized. However, in cases where environmental preferences are not phylogenetically conserved, or in cases where no environmental preference information is available for closely related taxa [54], predictions using taxonomic or phylogenetic information alone are unlikely to yield accurate inferences. Since genomic information can be generated across a broad range of taxa without the need for isolation and culturing, innovative genome-based models can be used to estimate the environmental preferences of those taxa (including multiple strains of a given species) that remain uncharacterized, revealing the specific traits underlying those preferences, and improving the accuracy of inferences derived from taxonomic and phylogenetic information.

Genome-based predictive models of environmental preferences need to be carefully designed and validated to be useful. Machine learning models are often used to infer phenotypic traits from genomic attributes because they can accommodate large numbers of nonlinear predictors such as genes or proteins expected to be associated with environmental preferences [55]. These models have been used to accurately predict diverse functional traits in bacteria such as metabolic capabilities [56] and have already been applied to predict the oxygen [57], temperature [46], pH [48], and salinity [58] preferences of bacterial taxa with available whole-genome information. The accuracy of machine learning models is often compromised by the typically small size of the training data (i.e. a limited number of taxa for which genomic data and direct measurements of environmental preferences are available) and data sparseness (i.e. many gene variants involved in environmental adaptation not being present in genomes selected for model training). Moreover, most available training data on environmental preferences are derived from readily culturable bacterial taxa, which often limits the generalizability of the predictions from these models. Several steps can be taken to minimize bias during model training and testing. For example, using “out-of-clade” model testing, machine learning model predictions are tested on taxa that are phylogenetically distinct from those used for model training, providing a fair evaluation of the general applicability of the model. Also, phylogenetically stratified crossvalidation can reduce model biases by maximizing the representativity of less frequent phylogenetic groups both during model training and testing [59]. Besides minimizing and accounting for phylogenetic biases, model predictions established using culture-based methods can be validated using environmental preference information obtained from culture-independent methods, or vice versa. In this manner, predictions that have been established using measured growth responses of isolates across controlled environmental gradients can be extended to novel taxa and lineages (Fig. 3).

## Coupling genomic and environmental preference information

Compared to metabolic or morphological traits, which are often predictable based on metabolic pathway completeness [60] or from the presence of selected genes [61], predicting environmental preferences often requires information on multiple traits. This makes environmental preferences particularly challenging to predict using genomic information alone, especially when we do not have *a priori* knowledge of which specific genes or other genomic attributes underly environmental adaptations. However, there is evidence that genome-based approaches can be effectively used to infer bacterial environmental preferences. For example, bacterial optimal growth temperature (OGT) has been predicted across a broad range of bacteria (>2500 cultured strains) using genome-derived features such as genome size and tRNA sequences [47], as well as using proteome-wide 2-mer amino acid composition [46]. Likewise, by pairing biogeographical information on taxon distributions with publicly available whole-genome data, a study used a machine learning model to infer the pH preferences of over 4500 unique taxa belonging to 38 different bacterial phyla across soil and freshwater systems [48]. Also, another recent study combined information on amino acid composition and the cellular localization of particular enzymes to infer with reasonable accuracy the oxygen, temperature, pH, and salinity preferences of genomes contained in the Genome Taxonomy Database (GTDB r214, >85 000 curated genomes) [58].

In some cases, genomic features can be selected *a priori* that are expected to be mechanistically linked to bacterial adaptations to particular environmental conditions. In the case of bacterial temperature preferences, amino acid composition impacts protein thermostability where thermophilic taxa are enriched in charged amino acids [62], and generally have a higher tRNA GC content [63]. Similarly, halophilic bacteria generally have higher proportions of acidic amino acids in the proteome to enhance protein stability [64]. In the case of oxygen preferences, the number of O_2_-utilizing enzymes [57] and the prevalence of residues susceptible to oxidation in amino acids [45] have been shown to be useful for inferring bacterial oxygen preferences.

Although the examples above rely on publicly available genomic data, obtaining metagenome-assembled genomes (MAGs) along environmental gradients can effectively overcome the taxonomic biases of reference genome databases, which tend to be skewed towards the subset of microbial taxa that are most readily cultured. For example, using metagenomic information from soils along aridity gradients, a study reconstructed 256 MAGs with distinct preferences for moisture availability, providing moisture preference information for taxa for which genomic data was not previously available [18]. This strategy can also identify novel genes underlying environmental preferences based on comparative genomics of MAGs or representative genomes of taxa along the environmental gradient. The functional analysis of MAGs along environmental gradients can also improve our understanding of intraspecific variation in environmental preferences, and several methodologies exist to obtain strain-level information from metagenomes ([65]). With sufficient genome coverage, analyzing MAGs along environmental gradients could even make it feasible to identify the environmental preferences and the specific genes (or other genomic features) underlying the environmental preferences for many taxa, which remain uncultivated, which would help elucidate their optimal isolation and cultivation conditions.

## Predicting bacterial environmental preferences from protein structures

Genomic information is only one of several sources of information that can be leveraged to improve our understanding of microbial environmental preferences. An approach that holds promise is the analysis of protein structures, based on the recognition that many protein properties are not directly predictable based on gene sequences. The amino acid composition of the proteome and the optimal folding of thermostable protein structures are key adaptations to temperature ([62]), and the pH at which a given protein fold is most stable positively correlates with the empirical subcellular pH [66]. This means the reconstruction of protein structures from amino acid composition can provide insights into some of the most likely environmental adaptations of bacteria. For example, since pressure impacts the folding and hydration level of proteins, piezophiles (taxa that are adapted to withstand high hydrostatic pressure) have a distinct amino acid composition and protein folding configuration of key enzymes [67]. Additionally, sets of genes used as predictive features could be refined by identifying which of those genes are under stronger selection, assuming those should be more strongly associated with a given environmental preference. Methods for the rapid reconstruction of protein structures are now available [68], making it feasible to use the structures of individual proteins to improve predictions of bacterial environmental preferences.

## Research priorities and best practices to expand our understanding of microbial environmental preferences

Scientists willing to quantify the environmental preferences of bacteria should not be discouraged by the myriad of limitations associated with all of the methods available, but embrace their integration. The key to expanding our knowledge of bacterial environmental preferences lies in the integration of culture-based and culture-independent approaches using methods that leverage bacterial genomic information. Quantifying the environmental preferences (and therefore the optimal growth conditions) for many currently uncultured taxa may promote the expansion of microbial culture collections. As we characterize novel taxa in culture, we concurrently improve the empirical data needed for genome-based model development, fostering a positive feedback loop. For example, the highly abundant marine bacterium *Candidatus Pelagibacter ubique* was first successfully cultivated in a defined medium whose composition was informed by genome-based metabolic models [69]. This breakthrough facilitated a breadth of studies that quantified the fundamental impact of this taxon on global biogeochemistry by coupling culture-based and culture-independent approaches, allowing the isolation of additional strains in the *SAR11* group and associated discoveries [70]. Genome-based predictions of environmental preferences could therefore accelerate the description of many functionally important taxa awaiting to be characterized, particularly when coupled with novel technologies for high-throughput cultivation. Unlocking this path will help reduce current biases in the phylogenetic distribution of environmental preference information, improve the determination of genes underlying environmental adaptation, and help quantify environmental preferences for relatively understudied factors (Fig. 1).

There is no single best approach to infer environmental preferences using genomic information. However, a set of recommendations emerge based on the challenges and pitfalls discussed in this piece. First, identify whether your environmental factor of interest exhibits a strong phylogenetic signal, which can save you the challenges of developing genome-based predictive models in the first place. If taxonomic or phylogenetic information is sufficient to accurately infer an environmental preference, no need to develop complicated models. Second, try diverse predictive features (both broad genomic/proteomic features and gene contents) to find the best performing set for your environmental variable of interest, and prioritize genes involved in environmental adaptation if such genes are known. Finally, assess the phylogenetic breadth of your training data, and validate your model on an independent dataset containing taxa from other clades, as this will determine the generalizability of model predictions. Microbial ecologists have the opportunity to expand our fundamental understanding of the environmental preferences of the vast majority of bacterial diversity that remains uncharacterized. The integration of traditional culture-based approaches with novel predictive approaches is the key to making it happen.

## Data Availability

The datasets analyzed during the current study are available in [29] (https://doi.org/10.6084/m9.figshare.c.4843290).
